# Determination of regulatory ionophore coccidiostat residues in feedstuffs at carry-over levels by liquid chromatography-mass spectrometry

**DOI:** 10.1371/journal.pone.0182831

**Published:** 2017-08-09

**Authors:** Loredana Annunziata, Pierina Visciano, Arianna Stramenga, Maria Novella Colagrande, Guido Campana, Giampiero Scortichini, Giacomo Migliorati, Dario Compagnone

**Affiliations:** 1 Istituto Zooprofilattico Sperimentale dell’Abruzzo e del Molise “G. Caporale”, Teramo, Italy; 2 Faculty of Bioscience and Technology for Food, Agriculture and Environment, University of Teramo, Teramo, Italy; 3 Istituto Zooprofilattico Sperimentale dell’Umbria e delle Marche, Perugia, Italy; Istituto di Biologia e Biotecnologia Agraria Consiglio Nazionale delle Ricerche, ITALY

## Abstract

In this study samples of feedstuffs were collected from different feed mills and animal farms located in central Italy and analyzed for ionophore coccidiostat residues at carry-over levels by liquid chromatography-mass spectrometry. Since unavoidable cross-contamination of feedstuffs may occur during their production as well as distribution and storage, the collection of samples covered all these different stages. Residues of lasalocid, monensin, salinomycin and maduramicin were detected in 32.4% of samples, both at production and storage level. The maximum content for unavoidable carry-over set by Regulation (EU) No 574/2011 was exceeded in 11.3% of samples. The variability of the results highlighted the different approach of each investigated feed business operator to avoid any cross-contamination in non-target feed. The method developed in this study can be able to detect ionophore coccidiostats at low concentrations consequent to carry-over.

## Introduction

Coccidiostats are pharmacologically active substances intended to kill or inhibit protozoa of the genus *Eimeria* causing a disease of the gastrointestinal tract in many farmed animals, such as chickens, turkeys, cattle, pigs, sheep and rabbits. They are authorized as feed additives for target animal species by the European legislation [1] and distinguished in polyether ionophores (lasalocid, monensin, maduramicin, narasin, salinomycin, semduramicin) produced by different bacteria, and non-ionophoric compounds (decoquinate, diclazuril, halofuginone, nicarbazin, robenidine) covering structurally diverse substances of synthetic origin [2]. Ionophore coccidiostats are defined as lipophilic chelating agents that transport cations across cell membranes, and this mechanism is not only efficient against coccidian, but it can act also against the mammalian cells. Some ionophoric compounds are highly selective for specific cations and can be monovalent (monensin, narasin, salinomycin, maduramicin and semduramicin) or bivalent (lasalocid). The mechanism of action of monovalent ionophores is based on entrapping monovalent metal cations (preferably sodium or potassium) and forming neutrally charged metal complexes, that can easily pass through prokaryotic and eukaryotic cell membranes [3,4], whereas lasalocid can directly translocate bivalent ions, such as calcium. These complexes can disrupt the normal trans-membrane ion gradient, causing energy reduction and cellular necrosis, which occurs primarily in cardiac and peripheral muscle cells [5]. Clinical signs of acute rhabdomyolysis have been observed in acute intoxications with monensin and other ionophoric compounds both in animals and humans [6].

The authorization of coccidiostats as feed additives is based on studies intended to demonstrate the safety of their use in relation to the target species at the highest proposed levels of incorporation in the feed or water and at a multiple of that level to establish a margin of safety [7]. However, when different types of feeds are produced within the same establishment and manufactured in the same production line, unavoidable traces can remain and contaminate the following feed production. This transfer is called carry-over or cross-contamination and may occur not only at all stages of feed production and processing, but also during transport and storage [8]. Carry-over represents a problem when traces of coccidiostats are present in feed for animal species in which their use is not authorized (referred to as non-target feed). Therefore, maximum levels (Table 1) have been established by Regulation (EU) No 574/2011 [9].

**Table 1:** Regulatory limits for ionophore coccidiostats in non-target feed following carry-over.

Compound	Non-target animal species	Maximum content (mg kg^-1^)*
**SEM**	laying birds and chickens	0.25
	other animal species	0.75
**LAS**	dogs, calves, rabbits, equine species, dairy animals, laying birds, turkeys and chickens	1.25
	other animal species	3.75
**SAL**	equine species, turkeys, laying birds and chickens, rabbits	0.7
	other animal species	2.1
**MON**	equine species, dogs, small ruminants (sheep and goat), ducks, bovine, dairy cattle, laying birds, chickens and turkeys	1.25
	other animal species	3.75
**NAR**	turkeys, rabbits, equine species, laying birds and chickens	0.7
	other animal species	2.1
**MAD**	equine species, rabbits, turkeys, laying birds and chickens	0.05
	other animal species	0.15

SEM = semduramicin; LAS = lasalocid; SAL = salinomycin; MON = monensin; NAR = narasin; MAD = maduramicin;

*relative to a feed with a moisture content of 12%.

A carry-over rate of approximately 3 and 1% of the authorized maximum content should be considered acceptable in feed for less sensitive and sensitive non-target animal species, respectively. Moreover, the carry-over rate of 1% should also be considered acceptable for cross-contamination of other feed for target species to which no coccidiostats are added, particularly for non-target feed for continuous food-producing animals. In fact, in dairy cows or laying hens there is evidence of transfer from feed to food of animal origin [10,11]. Thus, cross-contamination potentially results in exposure not only of non-target animals but also of consumers. Residues of coccidiostats in foodstuffs may represent a risk for human health because they could cause toxic effects, both directly in sensitive individuals, and indirectly for the promotion of resistant strains of bacteria [12].

Even if the feed business operators should take all appropriate measures during production, storage and transport of feeds containing the authorized coccidiostats to avoid any cross-contamination in non-target feed, the surveillance of this potential problem is indicated in the frame of official controls. Following Regulation (EC) No 882/2004 [13] the official controls must be carried out at any of the stages of production, processing and distribution both of feed and food, with an appropriate frequency. Few methods [12,14,15,16,17] have been published for coccidiostat determination at carry-over levels in animal feeds by liquid chromatography-mass spectrometry (LC-MS/MS). In the present study, a fast and reliable method was developed and used to investigate the potential cross-contamination of the regulatory ionophore coccidiostats (lasalocid, monensin, semduramicin, salinomycin, maduramicin and narasin) in feedstuffs collected during the official control plan carried out by the Italian competent authority.

## Materials and methods

### Sampling plan

In this study 71 feedstuffs (each sample was constituted of 500 g at minimum) were collected following Regulation (CE) No 152/2009 [18] amended by Regulation (EU) No 691/2013 [19] regarding the methods of sampling and analysis, and analyzed for the regulatory ionophore coccidiostats in the frame of official controls carried out during the years 2011–2015. The sampling of feedstuffs aimed specifically at investigating the potential carry-over as reported by Regulation (EU) No 574/2011 during different stages of the feed chain, and therefore it was carried out as follows: 26 and 16 samples were taken in the animal farms stored in unopened bags or bulk, respectively; 11 samples were collected directly from the manger or the feed distribution container; the remaining 18 samples were taken in feed mills from feedstuff lots produced just after the production of lots in which the addition of monensin was authorized for target species (S1 File).

### Reagents and standard solutions

Narasin (NAR), maduramicin ammonium alpha (MAD), nigericin sodium (NIG, used as internal standard) were supplied from Sigma (St. Louis, MO, USA). Salinomycin sodium (SAL), monensin sodium (MON), and only lasalocid sodium (LAS) as solution at 100 μg ml^-1^ in acetonitrile, were purchased from Dr. Ehrenstorfer GmbH (Augsburg, Germany). Semduramicin sodium (SEM) was kindly donated by European Union Reference Laboratory in Berlin.

The reagents used in this study were of HPLC or analytical grade and purchased from Sigma, except for ultrapure water that was supplied from Elga Labwater (Wicombe, UK). For each analyte (except for LAS) and for the internal standard, individual stock solutions at concentrations of 1000 μg ml^-1^ in methanol were prepared. A stock solution at 500 μg ml^-1^ in methanol was prepared only for SEM. All the solutions were stored below -18°C and remained stable for 3 months. Working standard solutions in acetonitrile were prepared daily for all the analytes (except for LAS, that was directly used as solution at 100 μg ml^-1^ in acetonitrile) just before the analysis. More in detail, a working standard solution containing MON at 62.5 μg ml^-1^, SAL and NAR at 35 μg ml^-1^, MAD at 2.5 μg ml^-1^, SEM at 12.5 μg ml^-1^, was prepared in acetonitrile after evaporating the methanol under nitrogen stream at 30°C. A working internal standard solution at 62.5 μg ml^-1^ was prepared in acetonitrile after evaporating the methanol under nitrogen stream at 30°C.

### Sample preparation and analysis

Grinded feed (5 g) was weighed into a polypropylene tube and then spiked with 100 μl of working internal standard solution at 62.5 μg ml^-1^ (corresponding to a spike of 1.25 mg kg^-1^). The sample was left for 30 min to improve standard dispersal in feed. Then, 50 ml of acetonitrile:methanol (90:10, v/v) were added and sample was shaken (1 h, 200 rpm) and centrifuged (2800*g* for 10 min). Finally, 1 mL of supernatant was transferred into an HPLC vial for instrumental analysis for SEM and MAD determination, while for LAS, MON, SAL and NAR, 1 ml of supernatant was diluted with 4 ml of acetonitrile before the instrumental analysis (dilution 1:5, v/v).

The chromatographic separation was obtained by a Perkin Elmer HPLC system (Perkin Elmer, Waltham, MA, USA). In detail, a microbinary pump model 200, an autosampler model 200 equipped with a degasser, and a column oven were used. A reversed-phase HPLC column (100 mm x 2.1 internal diameter, 3.5 μm) XTerra MS C18 Waters (Milford, MA, USA) with a guard column XTerra MS C18 Waters (10 mm x 2.1 mm internal diameter, 3.5 μm) was kept at 25°C during the analysis. The mobile phase, used in gradient mode at a flow rate of 0.2 mL min^-1^, was composed of a binary solvent system of acetonitrile:isopropanol (95:5, v/v) containing 0.1% formic acid (eluent A) and water:acetonitrile (95:5, v/v) containing 0.1% formic acid (eluent B). The gradient began with 80% of eluent A kept for 1 min and increased to 100% in 3 min, it was maintained for 6 min, decreased to 80% in 2 min, and equilibrated for 3 min. Then, 10 μl were used as volume of injection.

LC-MS/MS analysis was carried out by using an API 3000 triple quadrupole (Applied Biosystems, Toronto, ON, Canada) with an electrospray interface set in the positive ionization mode (ESI+) and operating in multiple reaction monitoring (MRM), by selection of one precursor ion and two product ions for each analyte. Table 2 showed the individual MRMs with their transition parameters. The ion source temperature was set at 450°C, while the capillary voltage at 5.5 kV. For the instrumental control and data processing the Analyst 1.4.2 software was used.

**Table 2:** LC-MS/MS parameters for detection of ionophore coccidiostats in MRM mode.

Analyte	Rt	Precursor ion (*m*/*z*)	Product ion (*m*/*z*)	DP (eV)	CE (eV)	CXP (eV)
**SEM**	5.3	895.5	833.3	45	43	17
			851.8	45	50	17
**LAS**	5.8	613.4	377.4	65	50	19
			357.2	65	66	9
**SAL**	6.3	773.6	431.2	80	50	11
			531.4	80	30	15
**MON**	6.6	693.5	479.3	110	70	8
			461.6	110	70	9
**NAR**	7.3	787.5	431.1	30	70	8
			531.1	30	62	10
**MAD**	7.5	939.5	877.5	55	40	21
			719.5	55	85	15
**NIG**	8.9	747.5	703.8	115	70	15

SEM = semduramicin; LAS = lasalocid; SAL = salinomycin; MON = monensin; NAR = narasin; MAD = maduramicin; NIG = nigericin; Rt = retention time; DP = declustering potential; CE = collision energy; CXP = collision cell exit potential.

The method was validated following the performance criteria of Regulation (EC) No 882/2004. The parameters into account were: instrumental linearity, specificity, limit of detection (LOD), limit of quantification (LOQ), precision, trueness, uncertainty. The instrumental linearity was evaluated by seven points calibration curves, containing the internal standard at 125 ng ml^-1^ for MAD and SEM, at 25 ng ml^-1^ for the other analytes. For each analyte, the curve concentration range was chosen to cover the three spiking levels tested in feed during the validation study. The linearity was tested for LAS, MON and SEM in the concentration range between 3.125–112.5 ng ml^-1^, for SAL and NAR between 1.75–63.0 ng ml^-1^, for MAD 0.63–22.5 ng ml^-1^. Moreover, 20 blank animal feed for different animal species (chicken, cattle, rabbit, swine) were analyzed to evaluate the specificity, LOD and LOQ.

For repeatability and reproducibility, samples of feed intended to chickens were spiked at half the lowest maximum limit (0.5 low-ML), the lowest maximum limit (low-ML) and the highest maximum limit (high-ML) fixed by Regulation (EU) No 574/2011. For each spiking levels three replicates were analyzed by two different operators, in two different occasions, for a total of 18 analysis. Trueness was expressed in terms of recovery (percentage of measured concentration versus fortified concentration) and precision (as relative standard deviation, %RSD). The accuracy and precision were evaluated using matrix-matched standards calibration curves consisted of blank sample subjected to the extraction procedure and fortified just before injection to obtain spiking levels at 0.5 low-ML, low-ML, high-ML.

After the addition of the working standard solution and the working internal standard at the end of the sample preparation, the sample was diluted with acetonitrile (dilution 1:5, v/v) only for LAS, MON, SAL and NAR.

From these two different validation days, the measurement uncertainty (MU) of the method was also determined and expressed as expanded uncertainty.

## Results and discussion

The aim of the development of our analytical method was to obtain a quick and efficient extraction of ionophore coccidiostats from feedstuffs at cross-contamination levels. Previous studies reported in literature demonstrated that acetonitrile could be used as extraction solvent for these compounds [12] or acetonitrile with sodium carbonate solution [14] or acetonitrile/water mixture [15], or ethanol [16] or methanol containing an acid or basic percentage [17]. In this study, the analytes were extracted directly from the matrix using an acetonitrile:methanol mixture (90:10, v/v) to obtain a clean extract without a clean-up step by solid phase extraction. Moreover, to overcome this interference issue, the sample was diluted with acetonitrile (1:5, v/v) only for LAS, MON, SAL and NAR determination, thus to maintain a good analyte signal and decrease matrix interference.

In comparison with the previously published studies, the described method showed some improvements. In particular, it was a fast and cheap method because it required only one extraction step instead of two as reported by Moretti et al. [16] and moreover, the sample extracts were not evaporated to dryness as mentioned in other papers [14,16], but they were directly injected for LC-MS/MS, for SEM and MAD, or they were diluted before the instrumental analysis, for LAS, MON, SAL and NAR.

The column and the chromatographic gradient allowed to obtain a good resolution for all the analytes. This is necessary for a quantitative detection of all the analytes.

LC-MS/MS conditions were optimized using individual analyte solutions at 10 mg l^-1^. The standards were injected directly in the mass spectrometer using a syringe pump operating with a flow rate of 10 μl min^-1^. The MS/MS fragmentation conditions were investigated and collision energies were optimized for each individual compound to give the required sensitivity. For all the analyte precursors two product ions were monitored following the performance criteria for mass spectrometry detection suggested in the Commission Decision (EC) No 657/2002 [20]. This approach allowed to achieve the identification points required by the mentioned Decision for the identification of these compounds.

The instrumental linearity was evaluated for all the analytes on seven calibration points, three replicates for point; the calibration levels were reported in Table 3.

**Table 3:** Calibration curve points.

Analyte	Concentration (ng ml^-1^)						
**SEM**	3.12	6.25	12.5	25.0	37.5	75.0	112
**LAS**	3.12	6.25	12.5	25.0	37.5	75.0	112
**SAL**	1.75	3.50	7.00	14.0	21.0	42.0	63.0
**MON**	3.12	6.25	12.5	25.0	37.5	75.0	112
**NAR**	1.75	3.50	7.00	14.0	21.0	42.0	63.0
**MAD**	0.625	1.25	2.50	5.0	7.50	15.0	22.5

SEM = semduramicin; LAS = lasalocid; SAL = salinomycin; MON = monensin; NAR = narasin; MAD = maduramicin.

**Fig 1:**
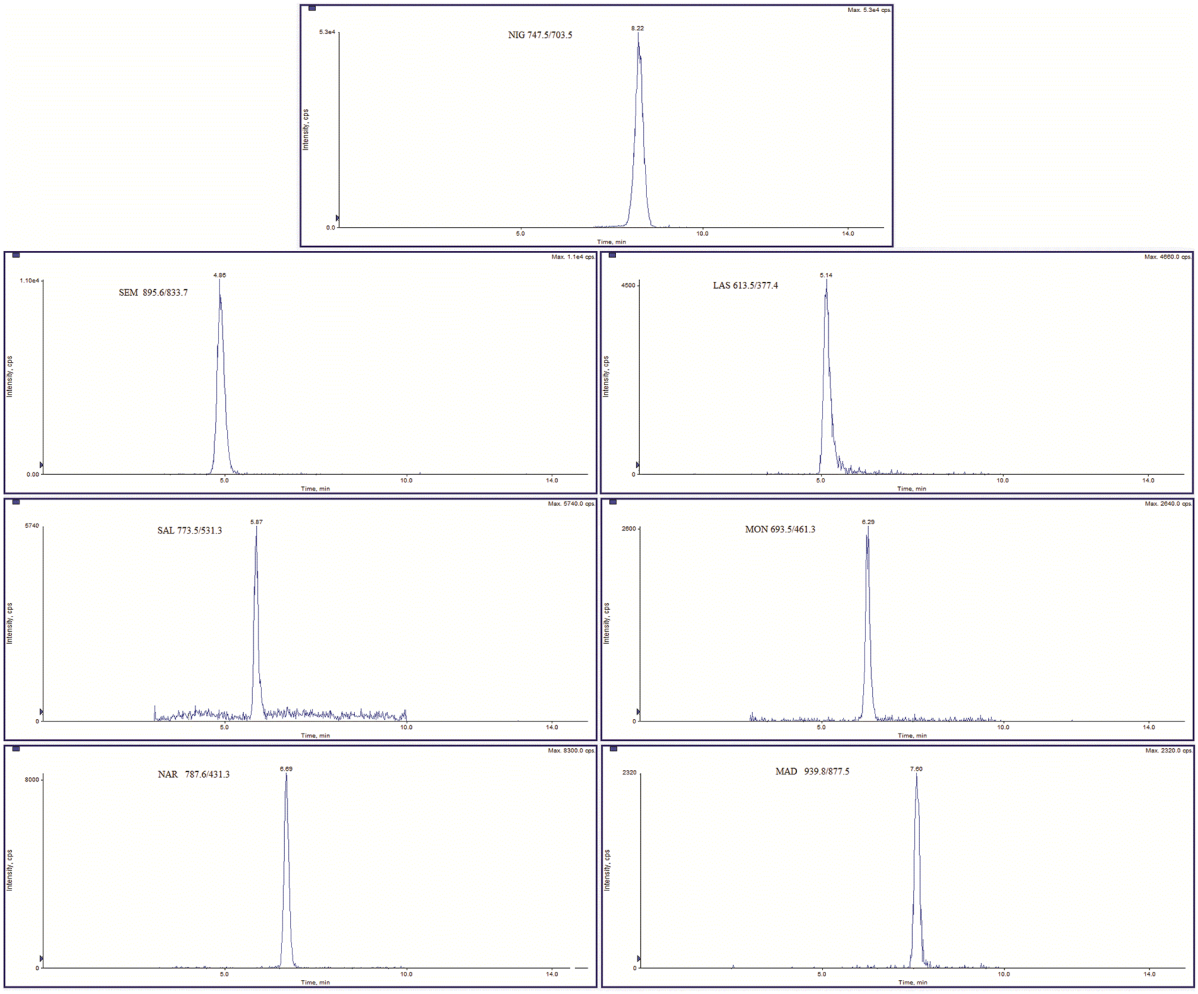
Chromatogram of standard solution at the following concentrations: Nigericin at 25 ng ml^-1^, semduramicin sodium and lasalocid sodium at 3.125 ng ml^-1^, salinomycin sodium at 1.75 ng ml^-1^, monensin sodium at 3.125 ng ml^-1^, narasin at 1.75 ng ml^-1^ and maduramicin ammonium at 0.625 ng ml^-1^. For each analyte, the most intense product ion was selected.

**Fig 2:**
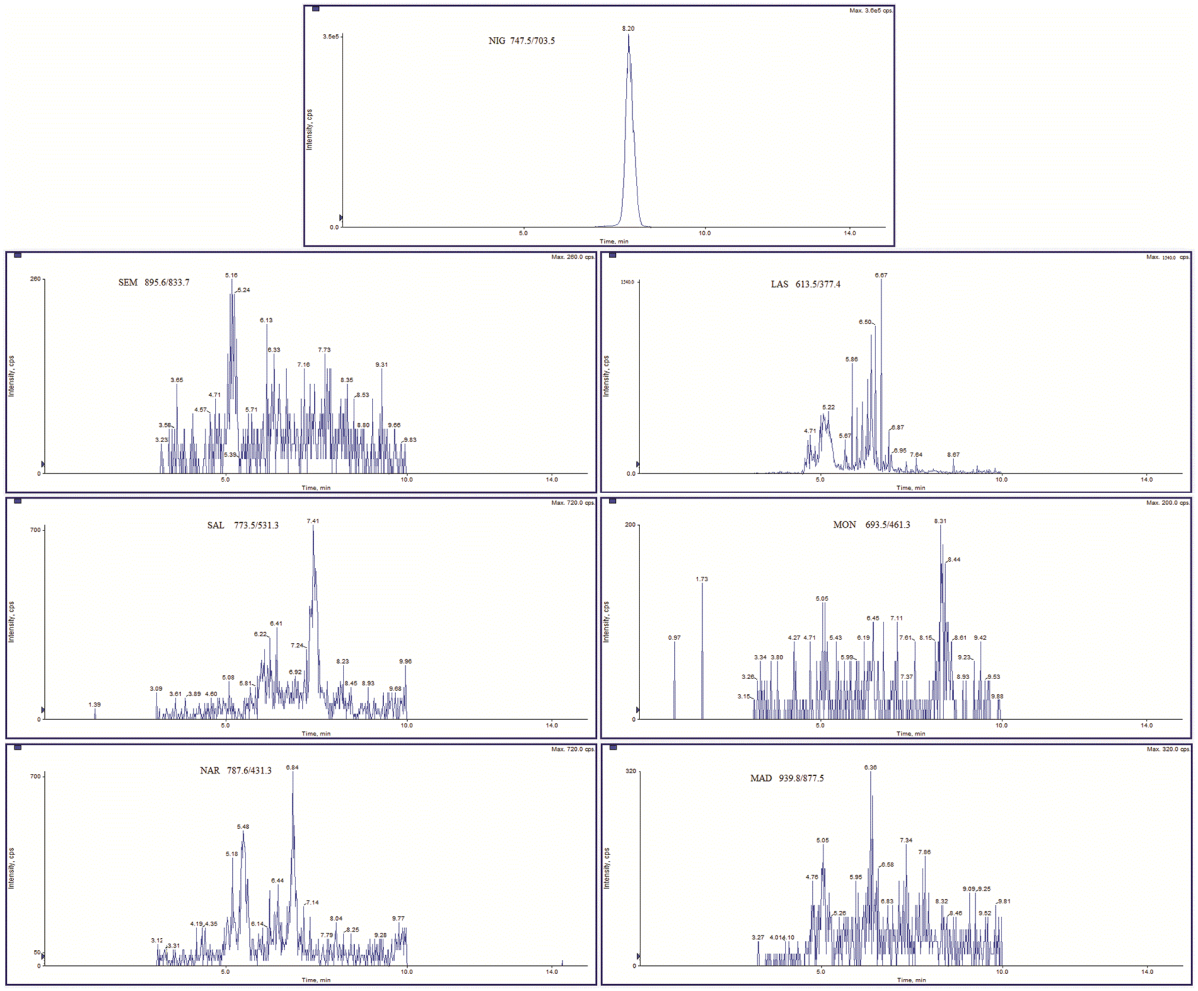
Chromatogram of representative blank feed spiked with 1.25 mg kg^-1^ of nigericin. For each analyte, the most intense product ion was selected.

Linearity was estimated by using the least square regression line equation. Calibration curves in solvent were constructed using the area ratio of the analyte peak to internal standard peak versus analyte concentration. The correlation coefficient indicated a good fit for all the analytes with values included in the 0.995–0.999 range (S2 File). A typical chromatogram of standard solution was reported in Fig 1, whereas Fig 2 showed the chromatogram of a representative blank feed.

To establish the specificity of the method, 20 blank feeds were analyzed in two validation days. All blank samples showed not interfering peaks in the retention time of interest for all the analytes.

These 20 blank feeds were used to evaluate LOD (i.e. three times of standard deviation of the noise signal of blank samples) and LOQ (i.e. six times of standard deviation). The obtained values for LOD and LOQ were below the target concentration for all the analytes, which proved that the developed method was sensitive enough to be used for the determination of ionophore coccidiostats at carry-over levels (Table 4).

**Table 4:** Validation data.

Analyte	LOD*	LOQ*	Spiking levels*	Recovery (%)**	Within reproducibility**	MU (%)
**SEM**	0.005	0.010	0.125	105.3	6.0	17.2
			0.250	96.6	5.2	
			0.750	90.4	4.6	
**LAS**	0.120	0.220	0.625	95.5	5.9	5.1
			1.25	95.1	5.0	
			3.75	86.5	4.7	
**SAL**	0.020	0.040	0.350	103.9	5.8	8.1
			0.700	103.1	2.7	
			2.10	87.7	2.9	
**MON**	0.060	0.110	0.625	101.0	3.4	9.1
			1.25	98.1	5.7	
			3.75	85.6	6.8	
**NAR**	0.030	0.060	0.350	107.3	3.7	10.1
			0.700	103.1	5.0	
			2.10	86.2	6.8	
**MAD**	0.002	0.005	0.025	102.7	13.4	10.2
			0.050	95.2	11.3	
			0.150	81.1	7.0	

LOD = limit of detection; LOQ = limit of quantification; MU = measurement uncertainty; SEM = semduramicin; LAS = lasalocid; SAL = salinomycin; MON = monensin; NAR = narasin; MAD = maduramicin;

*concentrations are expressed as mg kg^-1^;

** n = 6 replicates.

**Fig 3:**
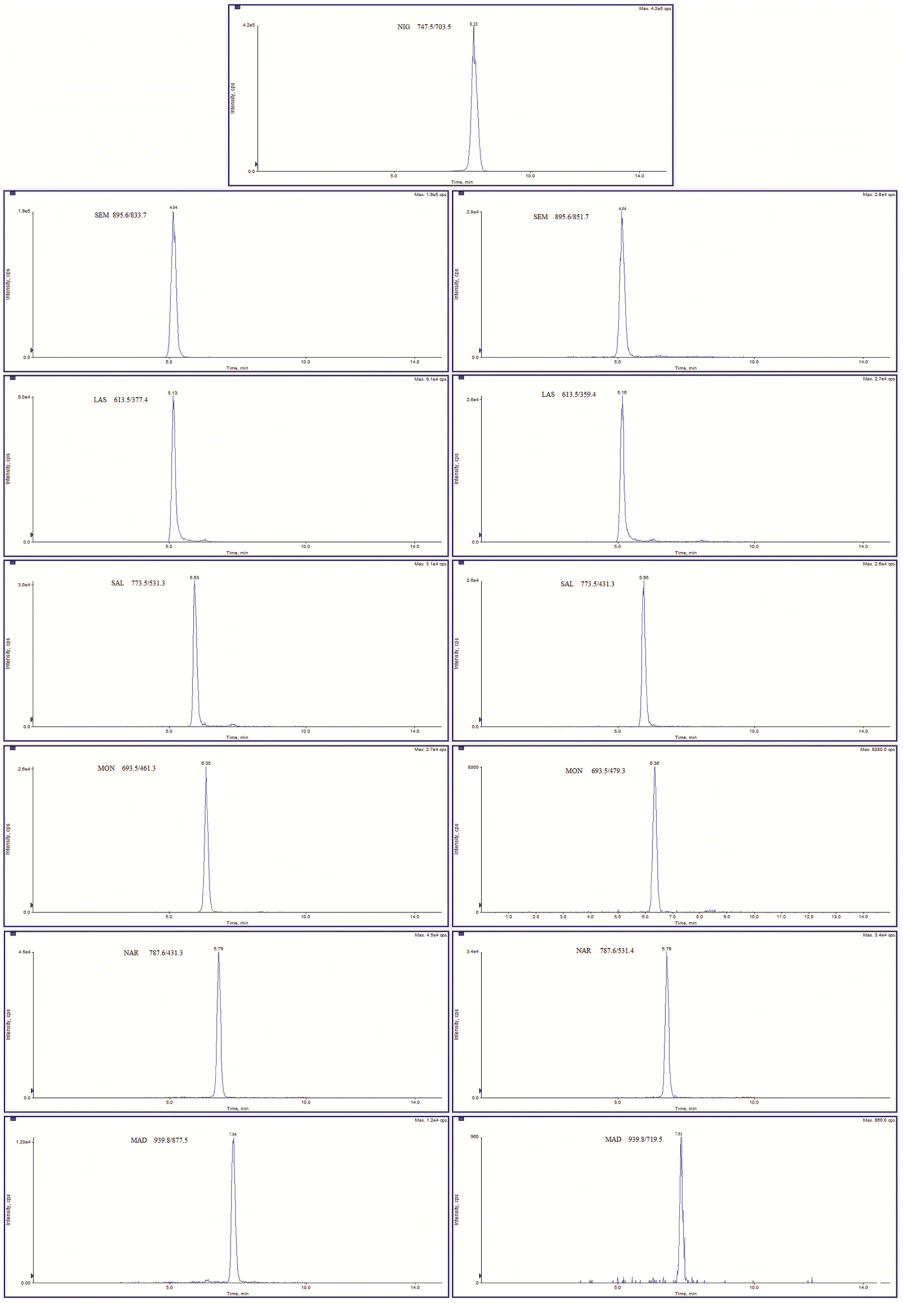
Chromatogram of representative blank feed spiked at the following concentrations: 1.25 mg kg^-1^ for nigericin, 0.125 mg kg^-1^ for semduramicin sodium, 0.625 mg kg^-1^ for lasalocid sodium, 0.350 mg kg^-1^ for salinomycin sodium, 0.625 mg kg^-1^ for monensin sodium, 0.350 mg kg^-1^ for narasin, 0.025 mg kg^-1^ for maduramicin ammonium.

Since no certified reference material was available, trueness was evaluated through recovery experiments. Repeatability and within-laboratory reproducibility were determined by analyzing spiking blank sample at three levels in two different days. The chromatogram of spiked feed at the lowest validation level were shown in Fig 3.

To minimize the matrix effects due to a straightforward sample preparation protocol, quantification was performed with matrix-matched standards calibration curves plotting the area of analyte divided the area of internal standard versus the concentration of analyte. The correlation coefficient indicated a good fit for all the analytes, with values included in the range 0.997–0.999.

Precision was expressed in terms of repeatability and within-laboratory reproducibility as %RSD. The results of quality assurance procedures were summarized in Table 4.

The mean recoveries for all the investigated compounds ranged between 81.1 and 107.3%. The maximum value of within-laboratory reproducibility was 13.4% for MAD at the spiking level of 0.025 mg kg^-1^. This value was the lowest %RSD calculated according Horwitz equation, in compliance with the requirement that %RSD should be as low as possible when the concentration levels are lower than 0.100 mg kg^-1^ [20].

For all the analytes, the measurement uncertainty was estimated considering the internal laboratory reproducibility. In detail, type A uncertainty was calculated from reproducibility measurements and matrix-matched calibration curve, whereas type B uncertainty derived from contributions due to balance calibration, micropipettes calibration, glassware uncertainty stated by manufacturer, and purity of reference materials stated on manufacturer’s certificate. The obtained value was multiplied by a coverage factor of two to have an expanded uncertainty calculated with a level of confidence of approximately 95%. This approach was recommended by the Eurachem/Citac Guide [21]. The obtained uncertainty values ranged between 5.1 and 17.2% (Table 4).

### Ionophore coccidiostat concentrations at carry-over levels

The concentrations of ionophore coccidiostats, referred to a moisture content of 12% as reported by Regulation (EU) No 574/2011, was reported in Table 5. The moisture content was calculated according to Regulation (CE) No 152/2009 [18] for positive samples. When sample concentrations resulted above the spiking levels (Table 4) further analyses were necessary to extend the matrix-matched standards calibration curve and verify the linearity of the applied method above to 1.5 times the obtained concentrations. Moreover, as all positive samples were analyzed in duplicate, MU was calculated taking in account the number of duplicates.

**Table 5:** Ionophore coccidiostat concentrations (mg kg^-1^ ± MU)* in feedstuffs at carry-over levels.

Sample	Target species	Sampling stage	MON	SAL	MAD	LAS
**1**	Chicken	Production line	3.47 ± 0.56**	-	-	-
**2**	Chicken	Unopened bag	-	0.311 ± 0.052	-	-
**3**	Chicken	Manger	-	0.085 ± 0.014	-	-
**4**	Swine	Feed distribution	0.760 ± 0.140	-	-	-
**5**	Rabbit	Manger	0.310 ± 0.060	-	-	-
**6**	Chicken	Bulk	0.140 ± 0.030	-	-	2.66 ± 0.27**
**7**	Chicken	Bulk	-	5.70 ± 0.83**	-	-
**8**	Cattle	Unopened bag	-	0.044 ± 0.007	-	-
**9**	Chicken	Manger	0.331 ± 0.061	-	-	-
**10**	Cattle	Unopened bag	-	2.47 ± 0.36**	-	-
**11**	Chicken	Production line	3.17 ± 0.70**	-	-	1.15 ± 0.13
**12**	Swine	Bulk	2.03 ± 0.37	-	-	-
**13**	Laying hens	Manger	0.359 ± 0.066	-	-	-
**14**	Rabbit	Manger	-	-	0.008 ± 0.002	0.574 ± 0.067
**15**	Swine	Feed distribution	-	0.816 ± 0.136	-	-
**16**	Swine	Unopened bag	1.46 ± 0.27	-	-	-
**17**	Chicken	Production line	4.17 ± 1.00**	-	-	-
**18**	Chicken	Production line	0.245 ± 0.045	-	-	-
**19**	Swine	Unopened bag	4.47 ± 1.07	-	-	-
**20**	Chicken	Production line	0.205 ± 0.038	-	-	-
**21**	Chicken	Production line	0.715 ± 0.132	-	-	-
**22**	Chicken	Unopened bag	-	-	0.395 ± 0.142**	-
**23**	Cattle, sheep and swine	Production line	5.79 ± 1.39**	-	-	-

MU = measurement uncertainty; MON = monensin; SAL = salinomycin; MAD = maduramicin; LAS = lasalocid;

*concentrations are expressed with 1 significant number if < 0.010; with 2 significant numbers in the range 0.010–0.100; with 3 significant numbers if > 0.100;

** samples exceeding the maximum limit.

Residues of MON, SAL, MAD and LAS were detected for a total of 23 (32.4%) samples. The maximum content for unavoidable carry-over set by Regulation (EU) No 574/2011 was exceeded in 8 (11.3%) samples. The most frequently detected coccidiostat was MON that exceeded the regulatory limit in 4 out of 18 samples collected in feed mills just after the production of feed lots added with MON. This result could be due to the unsatisfactory sanitation practices carried out on the production line. The other samples exceeding the maximum limit for SAL, MAD and LAS derived from storage in unopened bag or bulk in the animal farms. For feeds stored bulk in the animal farms, a failure in sanitation of feed containers or storage rooms could be supposed, whereas in case of positive samples collected from unopened bags the cross-contamination could previously occur during production or packaging, but it could not be demonstrated.

Few other studies reported coccidiostat residues in real samples of feed also in accordance with our results. In their study Moretti et al. [16] reported that monensin was the most detected compound (35%) in feed samples collected as part of the official Italian monitoring plans, followed by LAS (26%). The mean concentrations were 0.358 and 0.352 mg kg^-1^ for MON and LAS, respectively. Pietruk et al. [17] found ionophore residues in 72.7% of commercial feed, with more than one analyte in 18.2% of samples. Also, Delahaut et al. [14] detected coccidiostat concentrations above the 3% carry-over level for more than 15% of feed samples.

The general principles and requirements of food legislation indicate that the farm-to-fork approach consider feedstuffs as a sensitive stage at the beginning of the food chain, and feed business operators should take both preventive and corrective measures to ensure the safety of their product. The results of the present study allowed to suppose that cross-contamination of feedstuffs occurred not only at production level, but also during storage or distribution to animals. However, the lack of information about origin and/or production of all samples did not permit to assess for certain the source of cross-contamination since they were collected in the frame of the official controls and as such considered sensitive data.

## Conclusions

There is a strong need for analytical methods that enable the detection of ionophore coccidiostat residues also at low concentrations, both to allow the application of official controls and to check feed for compliance with legal provisions. The method developed in this study has been successfully optimized and validated for their determination in feedstuffs at carry-over levels. The results of the validation study confirmed satisfactory values for sensitivity, precision and trueness. The performed analyses of commercial feedstuffs confirmed the applicability of the method in the frame of the official controls.

## Supporting information

S1 File Sample origin. (XLSX)

S2 File Calibration curves. (DOCX)
