# Supplementary figures and images for: Determination of regulatory ionophore coccidiostat residues in feedstuffs at carry-over levels by liquid chromatography-mass spectrometry
Source: PLoS One. 2017 Aug 9;12(8):e0182831. doi: 10.1371/journal.pone.0182831 (PMC5549955; doi:10.1371/journal.pone.0182831)

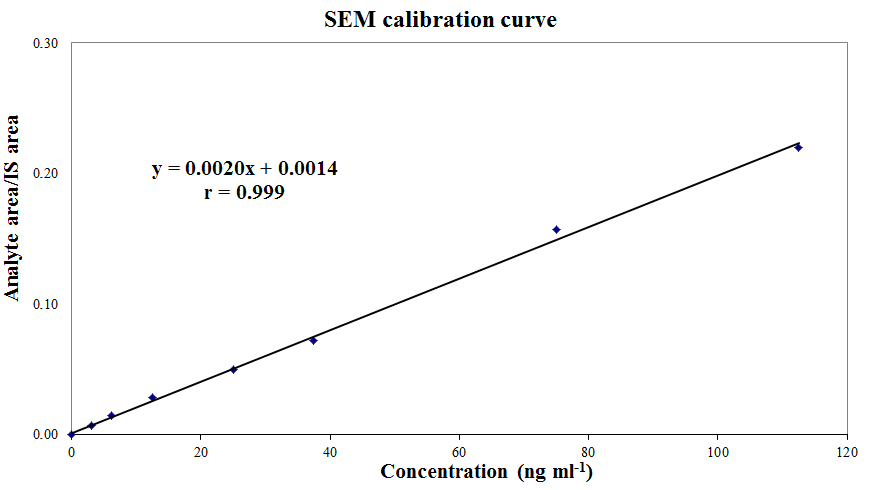


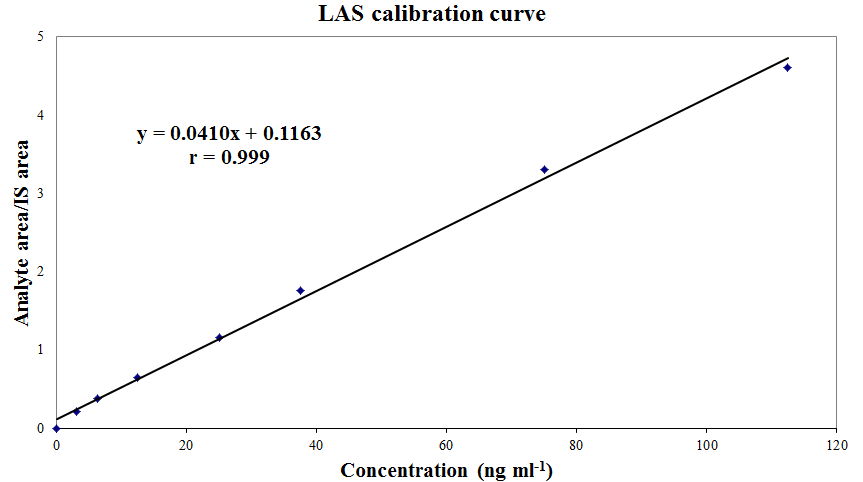


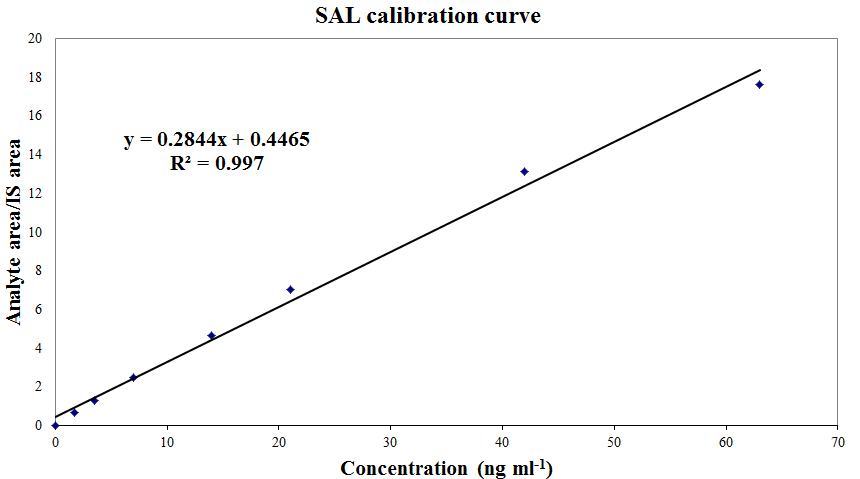


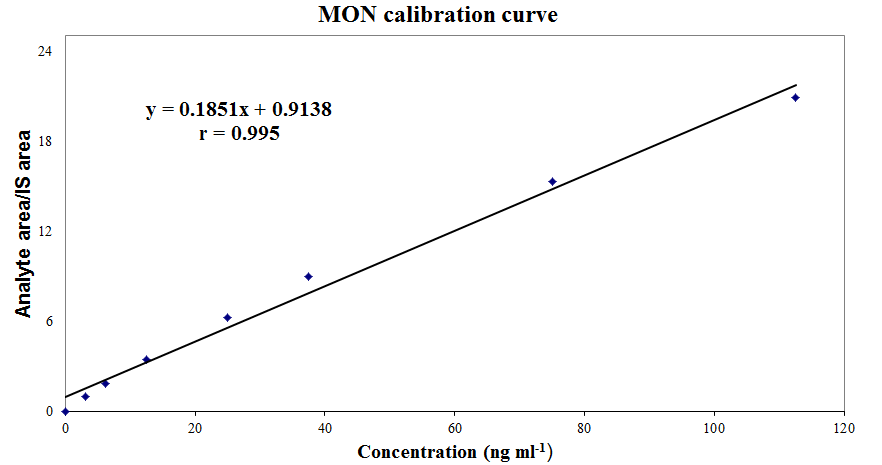


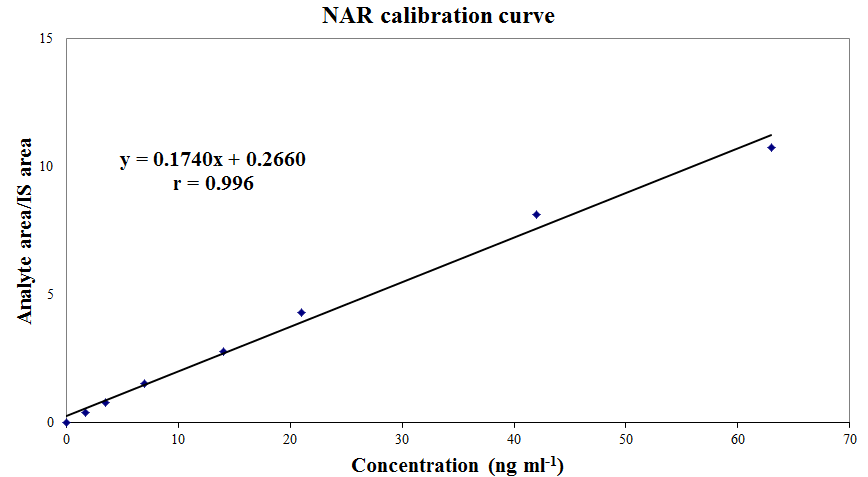


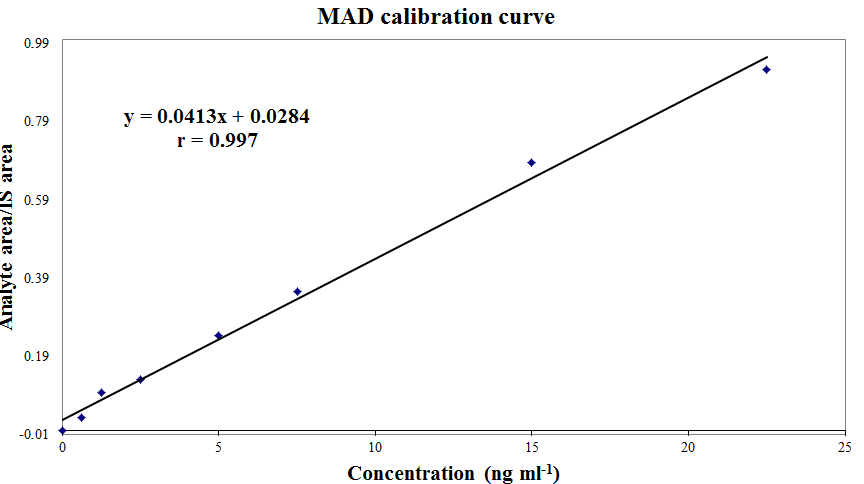

Supplement: S2 File — (DOCX) [file pone.0182831.s002.docx]
